# The metabolic landscape of tetrahydrobiopterin metabolism disorders in the Republic of Ireland

**DOI:** 10.1016/j.ymgmr.2024.101185

**Published:** 2024-12-31

**Authors:** A. Fisher, R. Boruah, P.D. Mayne, A.A. Monavari, E. Crushell, I. Knerr

**Affiliations:** a National Centre for Inherited Metabolic Disorders, Children's Health Ireland at Temple Street, Dublin, Ireland; b Department of Biochemistry, Children's Health Ireland at Temple Street, Dublin, Ireland

**Keywords:** Atypical PKU, Biopterin, DHPR deficiency, PTPS deficiency, Tetrahydrobiopterin

## Abstract

We present a case series of seven patients (5 males, 2 females, aged 7–38 yrs.) in Ireland with biopterin metabolism disorder. Five individuals had been diagnosed with dihydropteridine reductase (DHPR) deficiency and two with pyruvoyl tetrahydropterin synthase (PTPS) deficiency. While clinical symptoms were mainly neuro-developmental in nature, one of our patients with DHPR deficiency also had a mild pulmonary valve stenosis and patent arterial duct in infancy which subsequently resolved as a hitherto undescribed finding in this condition. Clinical outcomes in our patient cohort were overall satisfactory with the best outcomes in patients/siblings diagnosed on high-risk screening. In conclusion, early diagnosis, pathophysiology-driven treatments and frequent patient-specific treatment adjustments are crucial to sustain the best possible long-term outcomes.

Ireland's cohort of tetrahydropterin metabolism disorders highlights that improved outcomes are achieved with an early diagnosis which may not be attainable through newborn screening alone.

## Highlights

• The novel *QDPR* c.353C > T in our cohort was associated with mild neurodevelopmental impairment and epilepsy.
• One treated adult patient with DHPR deficiency successfully had three healthy children.
• Patients may be missed on current newborn screening programs. Early diagnosis is associated with improved outcomes.

## 1 Introduction

We here present outcome data for our patient cohort with tetrahydrobiopterin (BH4) deficiency, by and large identified among our patient cohort with phenylketonuria (PKU)/hyperphenylalaninaemia (HPA).

Disorders of phenylalanine (Phe) metabolism generally include classical Phenylketonuria (PKU), caused by profound deficiency of the enzyme phenylalanine (Phe) hydroxylase (PAH), and disorders of its cofactor system, such as tetrahydrobiopterin (BH4) defects. In healthy individuals, PAH catalyses the hydroxylation of Phe to tyrosine, using BH4 as its cofactor. Among the different metabolic fates of tyrosine is the production of neurotransmitters, such as dopamine [1,2].

As PKU is screened for in many countries worldwide, the diagnosis is typically established in the neonatal period. Approximately 1–2 % of all cases are considered to be caused by a defect of BH4, but disorders of the PAH cofactor system may still be underdiagnosed [3,4]. BH4 is an essential cofactor for Phe, Dopa and 5-OH-Tryptophan (5-HTP) metabolic pathways and also of nitric oxide synthases, increasing production of nitric oxide [5,6]. Disorders of BH4 synthesis or regeneration may mimic classical PKU or HPA; however, clinical symptoms and neurological sequelae can persist in these patients despite effective dietary control of blood Phe concentrations, e.g. due to altered serotonin and dopamine biosynthesis, if the diagnosis cannot be established early in life.

The National Bloodspot Screening Program (NBS) for (classical) PKU in Ireland was introduced in 1966. It also identified a very limited number of patients with atypical PKU due to BH4 deficiency, presenting biochemically similar to PKU/HPA patients. We previously described interim results for four patients with DHPR deficiency due to a hitherto undescribed pathogenic variant in the *DHPR* gene in the homozygous state [3]. All patients were treated with low Phe diet, Levodopa/Carbidopa, 5-HTP and folinic acid supplements.

We here present clinical outcome data for our entire cohort of patients with tetrahydrobiopterin metabolism disorders at the National Centre for Inherited Metabolic Disorders, together with biochemical findings, mutational spectrum and treatment approaches. This includes seven patients (5 males and 2 females) who had been diagnosed with either DHPR deficiency (*n* = 5), a defect in BH4 regeneration, or PTPS deficiency (*n* = 2), a defect in BH4 biosynthesis.

## 2 Methods

At the National Centre for Inherited Metabolic Disorders in Dublin we follow 7 patients with BH4 metabolism disorder (5 males and 2 females). We here describe their clinical findings and outcome, biochemical findings, mutational spectrum and treatment approaches using a retrospective chart review. Further to data collection descriptive statistics was applied.

## 3 Results

### 3.1 DHPR deficiency

Five patients with DHPR deficiency from three Irish families were diagnosed on NBS for PKU. Among those, two pairs are siblings, and the fifth patient does not appear to be related to the others based on a three-generation pedigree inspection. Patients with DHPR deficiency were diagnosed on NBS due to their raised blood Phe concentration (472–1608 μmol/L). Absent/deficient activity for DPHR was confirmed in these cases, along with altered blood pterins in dried blot spots, following further work-up of all neonates positive for newborn screening for PKU. The prevalent pathogenic variant was c.353C > T in exon 4 of the *QDPR* gene in a homozygous state in all our Irish patients with DHPR deficiency (*n* = 5). Treatment was started in the neonatal period once the diagnosis was made, including a low Phe diet and neurotransmitter replacement with Levodopa/Carbidopa, 5-HTP and folinic acid. The diet was generally commenced first, and neurotransmitter precursors were added as atypical PKU was diagnosed within the first month of life. CSF neurotransmitters, serum prolactin and blood Phe concentrations have been used for monitoring.

Symptoms ranged from none (asymptomatic due to early diagnosis and early commencement of treatment, including neurotransmitter precursors) to developmental delay and intellectual disability, movement disorder and epilepsy [Table 1]. Clinical symptoms included developmental delay, particularly speech and language delay/developmental deficits (60 %, or three patients), hearing loss (20 %, or 1 patient), tremor (20 %), seizures (20 %), abnormal neuroimaging demonstrating, e.g., sulcal dilatation with mild cerebral volume loss (40 %, or two adult patients). Furthermore, one female patient had a grade 2/6 ejection systolic murmur in the pulmonary area during infancy and was diagnosed with a mild valvar pulmonary stenosis and a small restrictive patent arterial duct which both gradually resolved. Clinical outcomes were overall satisfactory with the best outcomes seen in younger patients/siblings diagnosed on high-risk screening. At present, our patients with DHPR deficiency are 8–38 years of age (average 20 years, SD 13 yrs) and medically stable and well on their established treatments. Furthermore, our adult female with DHPR deficiency has three children who had normal growth parameters at birth and a normal NBS screening. During her pregnancies, her metabolic treatment included L-dopa/carbidopa (4–10 mg/kg/day), 5- HTP (5–8 mg/kg/day), folinic acid (10–15 mg/day) and she followed a Phe-restricted diet.

**Table 1:** Summery of clinical presentation and genetic data in our patient cohort.

	Age (yrs)	Gender	Enzyme defect and genotype	Age at diagnosis^1^	Phe at diagnosis (μmol/L)	Ethnicity	Clinical symptoms/outcome
Case 1	38	Female	**DHPR** <0.01 umol NADH/ m/gHb) (*QDPR* c.353C > T homozygous)	Newborn	>1000^2^	Irish traveller	Speech delay, motor delay/deficit, mild learning disability, epilepsy frequent falls, abnormal neuroimaging with mild cerebral atrophy, 3 healthy children
Case 2	36	Male	**DHPR** <0.01 umol NADH/ m/gHb) (*QDPR* c.353C > T homozygous)	Newborn	472	Irish traveller	Speech delay, mild learning disability, mild hearing loss, transient tremor, seizure, abnormal neuroimaging with very mild cerebral atrophy, medically stable and well.
Case 3	10	Male	**DHPR** <0.10 umol NADH/m/gHb) (*QDPR* c.353C > T homozygous)	Newborn	1608	Irish traveller	Sleep disturbance; no history of any abnormal movement or unsteady gait, medically stable and well.
Case 4	9	Female	**DHPR** <0.10 umol NADH/m/gHb) (*QDPR* c.353C > T homozygous)	Newborn	668	Irish traveller	Mild pulmonary valve stenosis and patent arterial duct in infancy- resolved. Normal development, intermittent convergent squint
Case 5	8	Male	**DHPR** <0.10 umol NADH/m/gHb) (*QDPR* c.353C > T homozygous)	Newborn	673	Irish traveller	Speech and language delay/deficit, mild general learning difficulty^3^
Case 6	7	Male	**PTPS** (*PTS* c.84-3C > G homozygous)	Newborn^4^	173	Roma	Normal development. Episodic lethargy with vertical deviation of both eyes occurring on different occasions – improved with dose increase of neurotransmitter precursors
Case 7	14	Male	**PTPS** (*PTS* c.84-3C > G homozygous)	3 years and 9 months	99	Roma	Motor delay; Speech and language delay, Oculogyric crises- responding to treatment

1 Newborns diagnosed by newborn screening bloodspot between 3 and 5 days of age.

2 Estimation by paper chromatography.

3 By Stanford-Bilet Intelligence Scales, Fifth edition.

4 Serum level; High risk screening.

### 3.2 PTPS deficiency

In our cohort of patients with PTPS (*n* = 2, two brothers aged 14 and 7 yrs.), the index case with PTPS deficiency was diagnosed at age 3 years and 9 months when he presented with developmental delay and acute oculogyric crises [Table 1]. It is unknown if this patient availed of a newborn screening in his home country, however it is notable that at the time of diagnosis his serum Phe level was only 99 μmol/L. His younger sibling was subsequently diagnosed as a newborn on high risk screening. Both patients with PTPS deficiency were found to be homozygous for the c.84-3C > G pathogenic variant in the *PTS* gene, a variant previously reported as disease-causing [7].

Our index case underwent a diagnostic oral Phe loading test using 100 mg/kg of Phe which revealed impaired Phe catabolism [8]. Phe increased from normal at baseline (91 μmol/L, Ref. 〈120) to 546 μmol/L 6 h after oral intake, indicating impaired hydroxylation in the liver. His blood biopterin results were below the cut-off of 5 nmol/L. The boy also had CSF neurotransmitters studies done which showed reduced concentrations for both homovanillic acid and 5-hydroxyindoleacetic acid (5-HIAA), indicating impaired catecholamine and serotonin metabolism. He was immediately commenced on L-dopa/Carbidopa and 5-HTP and the family had multidisciplinary team input and education. His blood prolactin level was tested after neurotransmitter replacement therapy commenced and was normal (prolactin 393, normal range 73–407 mU/L). The episodes of oculogyric crisis improved dramatically and have subsequently resolved. His EEG was normal on several occasions. He never required a low Phe diet. At age 9 he developed an episodic agitation which responded to an increased dose of neurotransmitter precursors. At age 14, he is doing well and is medically stable.

His younger brother was diagnosed as a neonate on high risk screening. His initial Phe level on metabolic screening was 173 μmol/L. Molecular genetic analysis revealed the same c.84-3C > G pathogenic variant in the *PTS* gene. Blood biopterin was low at <1 nmol/L. In CSF, neopterin was raised at 200 nmol/L (reference 7–65 nmol/L). He was noted to have a reduced concentration of 5-HIAA in CSF (148 nmol/L, Ref. 199–608 nmol/L) with a normal homovanillic acid concentration. The boy was commenced on 5-HTP and L-dopa/Carbidopa and did not require a low Phe diet in order to maintain normal blood Phe concentrations. His initial blood prolactin level was raised at 1863 mIU/L and subsequently normalised with sufficient neurotransmitter replacement therapy. His development was essentially normal. Prior to his regular medication dose increases, however, he experienced episodic lethargy with transient vertical deviation of both eyes.

Of note, our two patients with PTPS deficiency did not require any dietary restrictions to maintain essentially normal blood Phe concentrations. The patients are now aged 7 and 14 years of age and both medically stable and well.

## 4 Discussion

Disorders of BH4 metabolism are rare inherited metabolic disorders with approx. 1100 patients identified and tabulated in databases of paediatric neurotransmitter disorders [9]. In our centre, 7 patients have been identified to date which is approx. 1 % of our entire (classical) PKU patient cohort over the years.

Atypical PKU may mimic classical PKU with a raised blood Phe concentration on NBS; however, blood Phe may be only mildly raised in some cases [1]. The older patients with DHPR deficiency had generally more complex symptoms than the younger patients, i.e. their younger siblings, possibly due to advances of early diagnosis and commencement of treatment in the latter. Hence, early diagnosis and treatment seem to improve outcomes in patients with these rare disorders. While developmental delay, including speech and language delay, was a key finding in our cohort of patients with DHPR or PTPS deficiency, abnormal eye movements were only observed in our PTPS deficient patients.

Treatment consists of neurotransmitter supplementation, including Levodopa/Carbidopa, 5-HTP and, in DHPR deficiency, folinic acid and it may include a low Phe diet or the medication form of BH4 in order to control blood Phe concentrations [10]. In more detail, folinic acid is required as a secondary 5-methyltetrahydrofolate deficiency may occur in individuals with DHPR deficiency [10,11]. Furthermore, while the medication form of BH4 can enhance PAH activity in the liver and normalise blood Phe concentrations, there are patients with atypical PKU who do not respond to cofactor treatment. Therefore, neurotransmitter replacement therapy is essential, particularly as BH4 does not sufficiently cross the blood-brain-barrier [5,12].

Response to serotoninergic therapy can be monitored with CSF neurotransmitter studies, as clinically indicated, and the effect of dopaminergic therapy can be monitored with serial serum prolactin measurements [3,13]. Effective medication dose may vary considerably among patients [14], and Levodopa-refractory hyperprolactinemia may still be encountered in some individuals [15].

One of our patients with DHPR deficiency was diagnosed with a mild pulmonary valve stenosis and patent arterial duct in infancy which resolved spontaneously. Hitherto undescribed in patients with DHPR deficiency, it remains unclear as to whether altered activity of nitric oxide synthases and endothelial dysfunction, alongside altered inflammatory response, may have contributed to this feature [16].

In terms of pregnancy management and outcome, successful pregnancies with normal anthropometric values for her three children at birth was previously published for our adult female with DHPR deficiency [3,17].

Our patients with DHPR deficiency had raised blood Phe concentrations on NBS, as opposed to the children with PTPS deficiency. Therefore, in a population screened for classical PKU, atypical PKU may still be encountered in older children, e.g. with PTPS deficiency, as this may be missed by newborn screening. For instance, had our index case been screened in Ireland, levels similar to those encountered at presentation would have been considered a negative result (cutoff level 140 μmol/L at the time of his birth). This may be considered particularly for ethnic minority groups with a higher rate of consanguinity, such as the Roma population or the Irish traveller population, as presented here. Where clinical suspicion arises and BH4 levels are abnormal on metabolic testing, prompt evaluation with CSF neurotransmitter and targeted molecular-genetic analyses are required to allow for early and effective therapy in order to achieve the best possible outcomes. Molecular genetic diagnostics is required for all patients with persistently raised Phe, as treatments and outcome vary, depending on the underlying defect, including PAH, BH4, or more recently the co-chaperone DNAJC12 defects [18]. Furthermore, our case series reiterates the importance of measuring blood pterins, including DHPR activity, in patients with either a positive newborn screening result for PKU, or raised blood Phe concentration on selective metabolic testing, alongside confirmatory genetic testing. In a patient with hitherto unexplained neurological symptoms, such as movement disorders or oculogyric crises, biochemical metabolic work-up, including CSF studies, and targeted molecular-genetic testing can establish an underlying inborn genetic cause. This is in line with the diagnostic approach as outlined by the International Working Group on Neurotransmitter related Disorders iNTD [4].

In the Irish context, clinical outcomes in our cohort of patients with bioterin metabolism disorders are overall satisfactory. Taken together, early diagnosis and commencement of pathophysiology-driven treatments with sufficient neurotransmitter replacements are crucial, together with monitoring and frequent patient-specific therapeutic adjustments, in order to sustain the best possible neurological function and long-term outcome.

## Author contributions statement

IK conceived the study, overseen the clinical management part of the patient cohort and reviewed the manuscript. AF participated in the study planning and wrote the manuscript. RB, AAM and EC participated in patient management. PDM carried out and interpreted laboratory investigations.

## Informed consent

Written informed consent for the publication of this case report was obtained from the patients' legal guardians.

## Funding

This study was not supported by any funder.

## Ethics statement

All procedures were performed in compliance with the relevant laws and institutional guidelines. Institutional ethics and research approval was obtained prior to the study.

## Declaration of competing interest

The authors have no conflict of interest to declare.

## Data Availability

Data will be made available on request.
